# Etymologia: Blastomycosis

**DOI:** 10.3201/eid2011.ET2011

**Published:** 2014-11

**Authors:** Ronnie Henry

**Keywords:** etymologia, blastomycosis, Blastomyces dermatitidis, fungi, Gilchrist

## Blastomycosis [blasʺto-mi-koʹsis]

From the Greek *blastós* (“germ, sprout”) and *mykēs* (“fungus, mushroom”), this invasive fungal infection was first reported in 1894 by T. C. Gilchrist. Gilchrist initially believed the disease was caused by a protozoan, but in collaboration with W.R. Stokes, he subsequently isolated the organism, which he named *Blastomyces dermatitidis*. The infection became known as Chicago disease because most early cases were identified in the Chicago area, but it was subsequently shown to be endemic to much of eastern North America. Sporadic cases have also been reported in Africa, the Middle East, and India.

## References

[1] Chapman SW, Sullivan DC. *Blastomyces dermatitidis*. In: Mandell GL, Bennett JE, Dolin R, editors. Mandell, Douglas, and Bennett’s principles and practice of infectious diseases. 7th ed. Philadelphia: Elsevier; 2010. p. 3319–32.

[2] Dorland’s Illustrated Medical Dictionary. 32nd ed. Philadelphia: Elsevier Saunders; 2012.

[3] Saccente M, Woods GL. Clinical and laboratory update on blastomycosis. Clin Microbiol Rev. 2010;23:367–81 . 10.1128/CMR.00056-09 20375357 PMC2863359

